# Poster Session II - A193 EPIDEMIOLOGY OF ANTI-REFLUX MEDICATION USE IN VERY PRETERM INFANTS

**DOI:** 10.1093/jcag/gwaf042.193

**Published:** 2026-02-13

**Authors:** H Stevens, S Tanner, L Morrison, M Higgins, S Ghotra

**Affiliations:** Dalhousie University, Halifax, NS, Canada; Dalhousie University, Halifax, NS, Canada; IWK Health Centre, Halifax, NS, Canada; IWK Health Centre, Halifax, NS, Canada; IWK Health Centre, Halifax, NS, Canada

## Abstract

**Background:**

Gastroesophageal reflux disease (GERD) is common in preterm infants in the neonatal intensive care unit (NICU), documented in up to 22% infants <34 weeks gestation. Use of anti-reflux medications (ARMs) for GERD is not routinely recommended due to their lack of efficacy, as well as potential adverse effects. Despite this, ARMs continue to be prescribed in preterm infants. However, population based literature on prevalence, risk factors and long-term outcomes with ARM use in this group is scarce.

**Aims:**

1. To determine the prevalence and risk factors for ARM use during the first 6 months of life in very preterm infants.

2. To determine associations between early use of ARMs and long-term outcomes including hospital admissions, asthma treatments, and antibiotics in the first three years of life.

**Methods:**

A retrospective population-based cohort study was conducted, using the provincial Perinatal Follow-Up Program database. All infants born < 31 weeks gestational age during the period of 2005-2023 were screened. Infants with major congenital anomalies, neonatal deaths, or those lost to follow-up were excluded. Multivariable logistic regression was done to determine the association with long term outcomes, adjusting for gestational age and bronchopulmonary dysplasia (BPD).

**Results:**

A total of 952 infants were included. ARMs were prescribed in 445 infants (46.7%) in the first 6 months of life. About 2/3 of infants (292, 65.6%) were started on ARMs in NICU, and the remainder in the community after NICU discharge. Independent risk factors for ARM use included gestational age (per 1 week increase, OR 0.85, CI 0.78-0.92) and patent ductus arteriosus (OR 1.52, CI 1.07-2.14). There were higher odds of hospital re-admission and inhaled corticosteroid use in the ARM group (OR 1.5 CI 1.2-2; OR 1.9, CI 1.2-2.9). The odds remained significant when adjusted for gestational age and presence of BPD (Table 1).

**Conclusions:**

The high use of ARMs in the preterm population contradicts recent practice recommendations. ARM use is associated with increased risk of hospitalization and asthma in very preterm infants in the first three years of life, adding to the body of literature discouraging their use in this population.

**Funding Agencies:**

Dalhousie University Post Graduate Medical Education Office

**A193 Table 1:** Incidence of medical complications in the first 3 years of life, adjusted for gestational age and BPD

	No ARM use in 1st 6 months, n = 507	ARM use in 1st 6 months, n = 445	Adjusted Odds Ratio (95% CI)
Steroid inhaler use, n (%)	36 (7.1)	56 (12.6)	1.8 (1.14-2.83)^*^
Systemic steroid use, n (%)	16 (3.2)	22 (4.9)	1.45 (0.73-2.86)
Bronchodilator use, n (%)	117 (23.1)	133 (29.9)	1.29 (0.95-1.74)
Antibiotic use, n (%)	170 (33.5)	166 (37.3)	1.24 (0.94-1.63)
Re-admission to hospital, n (%)	166 (32.7)	190 (42.7)	1.34 (1.02-1.76)^*^

* p < 0.05

